# The impact of psychological stress on physiological indicators in healthcare workers: a cross-sectional study

**DOI:** 10.3389/fpubh.2024.1393743

**Published:** 2024-09-25

**Authors:** Na Li, Yan Wang, Yijiao Dong, Xiaoxue Chen, Bin Zhang, Xianghua Chen, Kejian Wang, Ying Sun

**Affiliations:** ^1^The Affiliated Hospital of Qingdao University, Qingdao, China; ^2^Innovative Institute of Chinese Medicine and Pharmacy, Shandong University of Traditional Chinese Medicine, Jinan, China; ^3^Systems Biology Research Center, Biology Institute, Guangxi Academy of Sciences, Nanning, China

**Keywords:** psychological stress, medical health workers, cross-sectional study, self-rating depression scale (SDS), physiological indicators

## Abstract

**Background:**

Medical health workers play an essential role in the healthcare system and face unique workplace stressors. However, the impact of psychological stress on their physical health has received less attention compared to the general population.

**Methods:**

We retrospectively analyzed the Self-rating Depression Scale (SDS) questionnaires and blood testing results from 1963 medical health workers. Multivariate linear regression analysis using a backward stepwise selection strategy to identify physical examination indicators that were significantly affected by depression.

**Results:**

Depression severity, as measured by SDS index score, was positively correlated with the levels of hemoglobin (coefficient 0.0027, *p* = 0.0412), platelet count (coefficient 0.0005, *p* = 0.0198), and uric acid (coefficient 0.0004, *p* = 0.0492), while negatively correlated with red blood cell count (coefficient-0.0895, *p* = 0.0406). Similar results were observed in the subgroup analysis stratified by age and sex.

**Conclusion:**

Our study found a significant association between higher levels of depression and specific physiological indicators in healthcare professionals, including elevated hemoglobin, platelet counts, and uric acid levels, as well as decreased red blood cell counts. These changes in blood parameters may reflect underlying physiological stress and inflammation, potentially increasing overall health risks for healthcare workers. Addressing these physiological changes may be crucial for mitigating the health risks associated with depression. To validate our findings and develop targeted interventions, larger multi-center studies are needed to further explore the relationship between depression severity and blood parameters in healthcare professionals.

## 1 Introduction

In the healthcare system, medical health workers play an essential role, yet they are subject to unique factors and multifaceted stressors that increase their susceptibility to psychological pressure (1, 2). These stressors are not only related to the high patient loads and complex, high-stakes medical decisions they must make but also include prolonged work hours, inadequate rest, and the emotional strain from regularly confronting patients’ suffering and mortality (3). The pressure to maintain precision in fast-paced environments, coupled with the ethical and legal responsibilities inherent in patients’ care, exacerbates these challenges (4). Moreover, healthcare workers often lack sufficient institutional support, further compounding their stress and potentially leading to burnout (5).

Given the intensity and persistence of these stressors, healthcare workers are at significant risk of chronic stress, which is increasingly recognized as a contributor to a range of physical health problems, such as cardiovascular disease, hypertension, and sleep disorders (6, 7). This chronic stress not only affects their well-being but also has implications for patient care quality, as stressed healthcare workers may experience decreased job performance and increased error rates (8). Therefore, investigating the psychological and physiological impact of stress on healthcare workers is crucial for both their health and the overall efficacy of healthcare systems.

Previous research has primarily focused on the psychological effects of stress, such as burnout, depression, and anxiety (9–11). However, the connection between psychological stress and physiological changes in healthcare workers remains underexplored, despite emerging evidence suggesting that chronic stress can lead to significant physiological alterations (12). For instance, psychological stress has been linked to changes in cardiovascular function, immune response, and metabolic processes, which could predispose individuals to long-term health issues (13–15). This gap in the literature highlights the need for further investigation into how stress affects specific physiological indicators in healthcare workers. For instance, Carpenter et al. found evidence that acute psychological stress and early-life adversity can impact the immune system by affecting the response of interleukin-6 (IL-6) in white blood cells (16). Another study reviewed the impact of psychological factors, such as stress, depression, social support, and optimism, on immune function and health (17). Although the effects of psychological stress on physical health have been extensively studied in the general population, relatively limited attention has been paid to its impact on medical staff, who are exposed to a unique set of stressors in the workplace.

Therefore, the objective of this study is to identify the key physiological indicators that are significantly affected by psychological stress among healthcare workers. Understanding the physiological manifestations of stress in healthcare workers is essential for developing comprehensive interventions that address both mental and physical health. By identifying specific physical examination indicators affected by psychological stress, we can gain insight into the broader health implications of stress in this population. These indicators can serve as objective measures to complement psychological assessments, offering a more holistic approach to evaluating and managing stress.

## 2 Methods

### 2.1 Participants

A retrospective study was performed by enrolling 1963 medical health workers, were from the Affiliated Hospital of Qingdao University, China. Participants’ sociodemographic information, including gender, age, educational level, marital status, and income, was collected through hospital records and direct interviews. Psychological and physical characteristics were assessed using standardized tools as described below. The data collection process was carried out by trained medical personnel to ensure consistency and reliability. The sample size was determined based on the total number of employees undergoing routine health check-ups at the hospital during the study period. The final sample size of 1963 was obtained after excluding individuals not suitable for inclusion. Participants meeting the following criteria were included in the subsequent data extraction and analysis: (1) aged over 18 years; (2) completed the health examination for employees at hospital, including blood tests and demographic information collection; (3) independently and voluntarily completed relevant psychological self-assessment questionnaires. To ensure the integrity and validity of the data collected for investigation, the exclusion criteria were as follows: (1) individuals with severe liver or kidney dysfunction; (2) individuals with hyperthyroidism or hypothyroidism; (3) those who declined to participate in the study or failed to complete the stress self-assessment questionnaire.

### 2.2 Psychological stress questionnaires

The study questionnaire was composed of two main components. The first part recorded the following information: sociodemographic characteristics such as age, gender, marital status, education level. The second part involved the Self-rating Depression Scale (SDS), which was a 20-item self-assessment scale based on the emotional, psychological and physical symptoms related to depression (18). Each item was scored from 1 to 4 on a 4-level scale. SDS index scores (ranging from 0 to 100) are classified as normal (< 50), mild depression (50 to 59), moderate to marked major depression (60 to 69), and severe to extreme major depression.

### 2.3 Blood test data

Hematological parameters, including white blood cell count, neutrophil count, neutrophil ratio, lymphocyte count, lymphocyte ratio, monocyte count, monocyte ratio, red blood cell count, hemoglobin, eosinophil count, eosinophil ratio, basophil count, basophil ratio, and platelet count were measured using the Sysmex XT-2000iV multiple automated hematology analyzer. Biochemical parameters, including glucose, uric acid, urea, creatinine, and calcium were determined by Hitachi 7,600 Automatic Biochemical Analyzer. All examinees are required to fast overnight before undergoing blood testing in the early morning.

### 2.4 Statistical analysis

The statistical analysis was conducted using SPSS (version 24.0, SPSS Inc., Chicago, IL) and R software (version 4.3.1, https://www.r-project.org/). Continuous variables were presented as mean ± standard deviation, while categorical variables were reported as numbers and percentages. The variability of demographic characteristics was compared using chi-square test and ANOVA. Multivariate regression analysis was performed to identify independent variables significantly associated with depression severity. A backward elimination procedure was carried out to iteratively removing the least significant variables until the lowest Akaike Information Criterion (AIC) was achieved. A *p*-value less than 0.05 was considered as the threshold for statistical significance.

## 3 Results

### 3.1 Basic characteristics of study subjects

In this study, a total of 1963 medical health workers were included, of whom 79.8% were female, with an average age of 34.74 years. Based on the SDS index scores (see Materials and Methods), participants were categorized into four groups according to the severity of depression (Table 1). Significant differences (*p* < 0.05) were observed in the proportion of female subjects, education level, income, systolic blood pressure, and red blood cell count among the individual SDS groups. Conversely, no notable inter-group differences were observed in age, marital status, BMI, diastolic blood pressure, and various blood test indicators (*p* > 0.05).

**Table 1:** Basic characteristics of the study participants.

	Total	SDS normal	SDS mild	SDS moderate	SDS severe	*p*-value
	*n* = 1963	*n* = 1,682	*n* = 208	*n* = 58	*n* = 15	
Age	34.74 (8.17)	34.84 (8.23)	34.57 (8.30)	32.93 (6.32)	32.93 (4.11)	0.271
Male (%)	397 (20.2)	359 (21.3)	33 (15.9)	5 (8.6)	0 (0.0)	0.006
Female (%)	1,566 (79.8)	1,323 (78.7)	175 (84.1)	53 (91.4)	15 (100.0)	
Marital status (%)						0.584
Married	1,478 (75.3)	1,274 (75.7)	152 (73.1)	39 (67.2)	13 (86.7)	
Unmarried	470 (23.9)	395 (23.5)	54 (26.0)	19 (32.8)	2 (13.3)	
Other	15 (0.8)	13 (0.8)	2 (1.0)	0 (0.0)	0 (0.0)	
Education (%)						<0.001
Middle school	10 (0.5)	8 (0.5)	2 (1.0)	0 (0.0)	0 (0.0)	
High school	4 (0.2)	2 (0.1)	2 (1.0)	0 (0.0)	0 (0.0)	
Junior college	153 (7.8)	116 (6.9)	31 (14.9)	4 (6.9)	2 (13.3)	
Undergraduate	1,256 (64.0)	1,062 (63.1)	136 (65.4)	46 (79.3)	12 (80.0)	
Master	349 (17.8)	316 (18.8)	28 (13.5)	5 (8.6)	0 (0.0)	
PhD	191 (9.7)	178 (10.6)	9 (4.3)	3 (5.2)	1 (6.7)	
Income by RMB (%)						<0.001
<1,000	20 (1.0)	17 (1.0)	0 (0.0)	2 (3.4)	1 (6.7)	
1,000–2000	25 (1.3)	18 (1.1)	5 (2.4)	1 (1.7)	1 (6.7)	
2,000–3,000	18 (0.9)	11 (0.7)	7 (3.4)	0 (0.0)	0 (0.0)	
3,000–5,000	169 (8.6)	129 (7.7)	28 (13.5)	12 (20.7)	0 (0.0)	
5,000–10,000	950 (48.4)	809 (48.1)	102 (49.0)	29 (50.0)	10 (66.7)	
10,000–15,000	594 (30.3)	525 (31.2)	53 (25.5)	13 (22.4)	3 (20.0)	
15,000–20,000	64 (3.3)	57 (3.4)	6 (2.9)	1 (1.7)	0 (0.0)	
>20,000	123 (6.3)	116 (6.9)	7 (3.4)	0 (0.0)	0 (0.0)	
BMI	23.29 (3.59)	23.35 (3.62)	22.75 (3.25)	23.43 (3.77)	22.94 (2.45)	0.141
SBP	119.94 (12.32)	120.19 (12.18)	118.95 (13.38)	118.34 (11.41)	111.93 (12.67)	0.025
DBP	73.33 (9.47)	73.46 (9.50)	72.84 (9.50)	72.07 (8.38)	69.53 (8.01)	0.226
WBC count	6.07 (1.59)	6.07 (1.61)	6.00 (1.52)	6.26 (1.56)	6.20 (1.01)	0.727
Neutrophil count	3.36 (1.24)	3.36 (1.25)	3.33 (1.20)	3.50 (1.21)	3.47 (0.80)	0.804
Neutrophil percentage	54.50 (8.41)	54.46 (8.48)	54.62 (7.96)	55.08 (8.23)	55.95 (8.44)	0.847
Lymphocyte count	2.16 (0.59)	2.16 (0.60)	2.13 (0.55)	2.21 (0.57)	2.19 (0.60)	0.793
Lymphocyte percentage	36.41 (7.84)	36.44 (7.90)	36.32 (7.53)	35.97 (7.23)	35.33 (8.10)	0.915
Monocyte count	0.39 (0.12)	0.39 (0.12)	0.39 (0.12)	0.39 (0.10)	0.39 (0.11)	0.969
Monocyte percentage	6.53 (1.65)	6.53 (1.67)	6.61 (1.50)	6.50 (1.73)	6.31 (1.02)	0.854
RBC count	4.61 (0.44)	4.63 (0.44)	4.52 (0.40)	4.56 (0.33)	4.59 (0.32)	0.011
Hemoglobin	135.43 (14.30)	135.63 (14.53)	134.19 (13.17)	133.79 (11.30)	136.53 (12.67)	0.433
Eosinophil count	0.12 (0.10)	0.12 (0.10)	0.11 (0.08)	0.12 (0.07)	0.12 (0.09)	0.734
Eosinophil percentage	1.99 (1.54)	2.01 (1.58)	1.88 (1.32)	1.90 (1.10)	1.89 (1.31)	0.682
Basophil count	0.03 (0.02)	0.03 (0.02)	0.03 (0.02)	0.03 (0.01)	0.03 (0.02)	0.906
Basophil percentage	0.57 (0.29)	0.57 (0.29)	0.57 (0.28)	0.54 (0.17)	0.52 (0.32)	0.846
Platelet count	256.90 (56.89)	256.17 (57.41)	257.92 (52.69)	273.47 (48.72)	260.33 (76.62)	0.15
Glucose	4.95 (0.66)	4.95 (0.65)	4.96 (0.77)	4.90 (0.48)	4.74 (0.46)	0.596
Uric acid	295.71 (80.27)	296.14 (80.54)	292.71 (79.06)	285.15 (73.51)	329.13 (89.09)	0.268
Urea	4.77 (1.53)	4.78 (1.59)	4.66 (1.14)	4.85 (1.03)	4.47 (0.75)	0.617
Creatinine	81.27 (24.53)	81.52 (26.10)	80.21 (12.05)	77.89 (8.53)	80.71 (5.37)	0.642
Calcium	2.40 (0.09)	2.40 (0.09)	2.40 (0.10)	2.40 (0.09)	2.39 (0.12)	0.867

SBP, systolic blood pressure; DBP, diastolic blood pressure; WBC, white blood cell; RBC, red blood cell.

### 3.2 Multivariate regression analysis

Regarding the four levels of SDS index score, multivariate regression was conducted with physical examination indictors utilized as independent variables and sociodemographic characteristics included as covariates. By employing the backward elimination procedure (see Materials and Methods), nonsignificant independent variables were iteratively removed that led to an optimal regression model (Table 2). Upon obtaining the final output, it was observed that levels of hemoglobin (Coefficient: 0.0027, 95% CI: [0.0001,0.0054], *p* = 0.0412), platelet count (Coefficient: 0.0005, 95% CI: [0.0001,0.0009], *p* = 0.0198), and uric acid (Coefficient: 0.0004, 95% CI: [0.0000,0.0007], *p* = 0.0492) demonstrated positive correlations with depression severity. Conversely, red blood cell count exhibited a negative correlation (Coefficient: -0.0895, 95% CI: [−0.1750, −0.0039], *p* = 0.0406). Our results indicate that higher levels of hemoglobin, platelet count, and uric acid are significantly associated with increased depression severity. Conversely, a lower red blood cell count is significantly associated with reduced depression severity. Meanwhile, we found that covariates, such as education category (Coefficient: -0.0469, 95% CI: [−0.0809, −0.0130], *p* = 0.0068) and income category (Coefficient: -0.0420, 95% CI: [−0.0641, −0.0198], *p* = 0.0002), are negatively correlated with depression severity.

**Table 2:** Significant variables associated with depression severity in multivariate regression.

		Coefficient	(95% CI)	*P*-value
Independent variables	SBP	−0.0021	(−0.0041, −0.0001)	0.0374
	RBC Count	−0.0895	(−0.175, −0.0039)	0.0406
	Hemoglobin	0.0027	(0.0001, 0.0054)	0.0412
	Platelet count	0.0005	(0.0001, 0.0009)	0.0198
	Uric acid	0.0004	(0.0000, 0.0007)	0.0492
Covariates	Age category	0.0254	(−0.0145, 0.0653)	0.2121
	Sex	0.0744	(−0.0081, 0.1569)	0.0772
	Marital Status	0.0016	(−0.0584, 0.0617)	0.9573
	Education category	−0.0469	(−0.0809, −0.0130)	0.0068
	Income category	−0.0420	(−0.0641, −0.0198)	0.0002

### 3.3 Subgroup analysis

**Figure 1:**
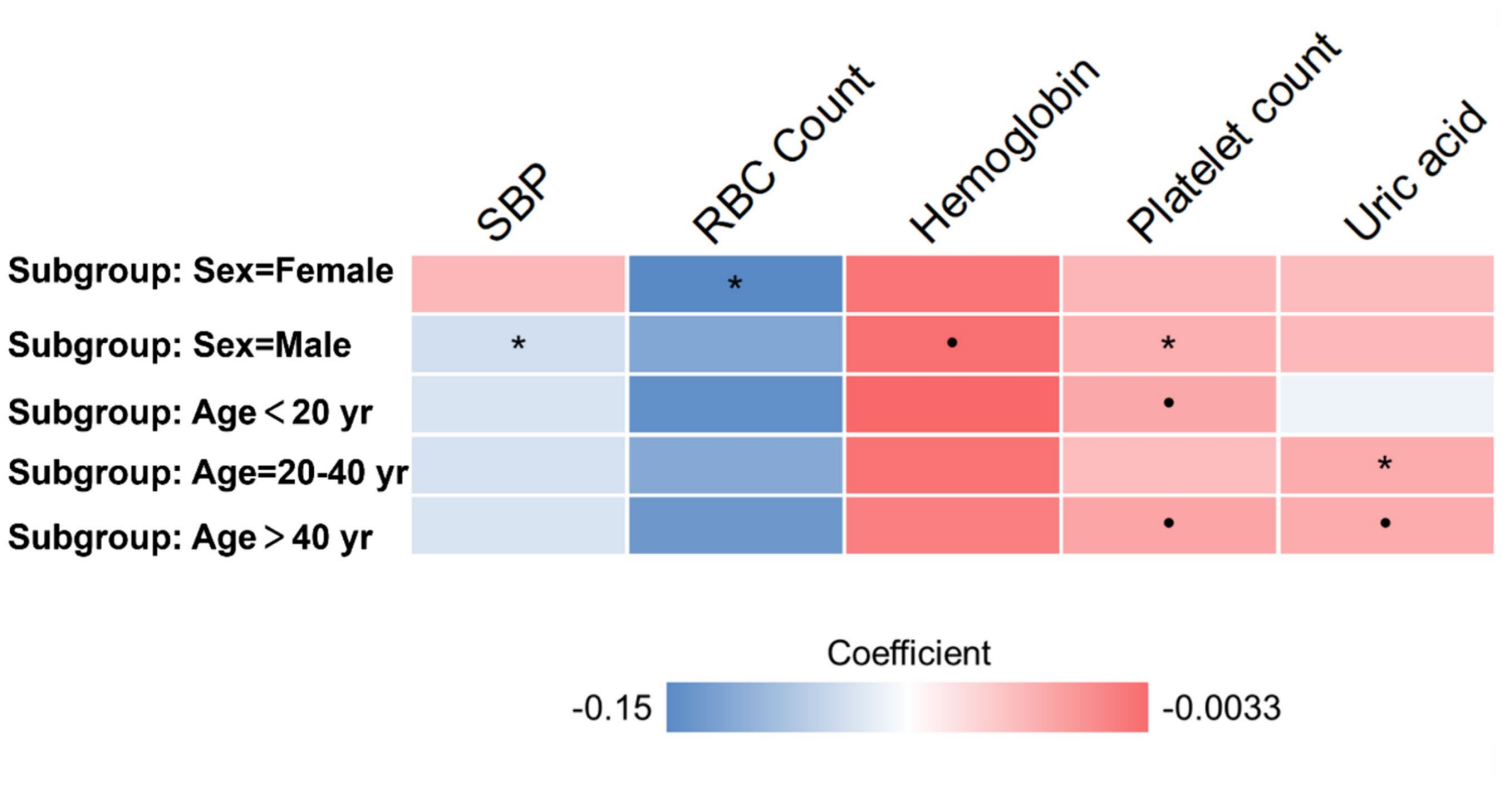
Subgroup analyses based on age and sex. Heatmap presents the coefficient in multivariate regression. The • mark indicates *p* < 0.1. The * mark indicates *p* < 0.05.

We conducted further subgroup analyses on the same cohort, dividing participants based on age and sex. The age subgroup analysis involved categorizing participants into three groups: young (below 30 years), middle-aged (30 to 40 years), and older (over 40 years) individuals. Similarly, the sex subgroup analysis involved segregating participants into male and female categories. In multivariate regression analysis on each subgroup, we observed consistent positive or negative correlations between depression severity and physical examination indictors (Figure 1). In females, SBP is negatively correlated with depression severity, while platelet count is positively correlated with depression severity. In males, RBC Count is negatively correlated with depression severity. In the age group of 20–40 years, uric acid is positively correlated with depression severity. However, due to the smaller sample size of subgroups, some of the observed correlations did not reach statistical significance (*p* > 0.05).

## 4 Discussion

This study sought to bridge the existing research gap by investigating the impact of depressive symptoms on the physical health of medical staff, a topic that has received relatively limited attention. While prior studies have extensively examined the association between psychological distress and physical health within the general population, the unique experiences of medical staff, who encounter distinct stressors in their work environment, remain understudied. The observed associations between depressive symptoms and specific physical health indicators, such as elevated uric acid and platelet levels, underscore the potential physiological consequences of psychological stress in healthcare workers. This highlights the need for more nuanced approaches to mental health assessments, where physical health indicators could serve as complementary markers of psychological distress. For instance, the interplay between oxidative stress and depression, as evidenced by the elevated uric acid levels, may offer novel insights into how chronic stress affects metabolic pathways.

In our study, we observed a significant positive correlation between depression severity and uric acid levels in the blood of healthcare workers. This finding is consistent with previous research that highlights elevated serum uric acid levels in individuals with depression (19–21). Specifically, studies have demonstrated that psychological distress is often associated with increased oxidative stress and lipid peroxidation, which can elevate uric acid levels (22). This aligns with our results, reinforcing the notion that uric acid may serve as a biomarker for psychological conditions. The convergence of our findings with the existing literature suggests that uric acid’s role in depression could be a reflection of its involvement in oxidative stress pathways.

From a biological perspective, depression-induced low mood may facilitate lipid peroxidation, contributing to increased oxidative damage. This oxidative stress can enhance nucleic acid degradation, subsequently elevating serum uric acid levels (23). These mechanisms underscore the importance of considering uric acid as a potential biomarker for psychological stress among healthcare workers. Furthermore, integrating our findings with research on the interplay between psychological disorders and metabolic pathways highlights the necessity for further interdisciplinary studies to explore these associations in depth (24, 25).

Our study also found a positive correlation between depression severity and platelet levels. This observation is supported by existing research, which suggests that depression and anxiety can activate the sympathetic nervous system, influencing platelet activity and production (26). Furthermore, the role of the immune system in both depression and platelet regulation warrants investigation, particularly in light of emerging research on the interactions between depressive symptoms and immune function (27, 28). Additionally, these findings hold significant clinical implications. Monitoring platelet levels may offer valuable insights into the physiological manifestations of depression (29, 30), potentially aiding in the early identification and management of depression-related issues among medical health workers.

We identified a positive correlation between higher depression severity and increased hemoglobin levels, as well as a negative correlation with red blood cell count. This seemingly contradictory discovery may have rational explanations, which include the following three possibilities: (1) Depression may lead to the breakdown of red blood cells, resulting in the release of hemoglobin into the bloodstream. This could cause an elevation in hemoglobin levels while decreasing the overall count of intact red blood cells. (2) Depression may cause a transfer of red blood cells from peripheral circulation to specific organs or tissues, thereby reducing their total count, but increasing the levels of hemoglobin. (3) The body may respond to depression by compensating for the decreased red blood cell count through increased production of hemoglobin. (4) The relationship between hemoglobin levels and depression severity may be influenced by other unmeasured factors, such as nutritional status, hydration levels, or the presence of chronic inflammatory conditions. Additionally, the observed correlations might be confounded by external stressors unrelated to occupational factors, suggesting the need for more comprehensive assessments that account for these variables in future research. These explanations align with existing research on the physiological responses to depression and provide insight into the complex interactions between depression and hematological parameters (31).

Our study has important implications and contributions in the field of healthcare (32). Firstly, we have identified specific physical examination indicators that may be influenced by depressive symptoms among medical staff, thereby contributing to their overall health and welfare (33). The integration of these biomarkers into routine health evaluations can potentially transform how depression is managed among healthcare professionals. Early identification through these indicators allows for timely, targeted interventions that not only address mental health symptoms but also prevent the potential downstream physiological consequences. This approach could reduce the burden of disease associated with untreated depression and improve overall workforce health and productivity. Secondly, the recognition of these impacts enhances patient care, as appropriate measures can be implemented to effectively address and manage depression, leading to improved health outcomes. Offering access to counseling services, stress management programs, and mental health resources not only addresses current mental health concerns but also helps prevent the escalation of depressive symptoms. Thirdly, the findings of our study can inform the development of preventive strategies aimed at reducing depression levels among medical staff (34). By taking proactive measures to assist medical professionals in managing depression before it adversely affects their health, we can promote their well-being. Additionally, our study provides a valuable foundation for future research in this area, which can explore the underlying mechanisms and evaluate targeted interventions to enhance the well-being of medical professionals.

An important advantage of studying medical staff with less occupational heterogeneity is the increased precision and validity of assessing the impact of specific occupational factors on health outcomes. As medical staff share similar occupational exposures and work-related risks, the homogeneity of this population allows for more accurate evaluations of the effects of occupational factors on health. This unique advantage enhances the internal validity of the study findings, providing robust evidence for the associations between occupational factors and health outcomes. By minimizing the confounding effects of diverse occupations, research on medical staff can provide valuable insights into targeted interventions and policies in the field of occupational health.

This study also has several limitations that need to be acknowledged. Firstly, the retrospective study design imposes inherent constraints that make it difficult to completely avoid selection bias, potentially distorting our findings. This design also restricts our ability to establish temporality, making it difficult to determine whether the observed correlations are causal or simply associative. For example, while we found significant correlations between depression severity and certain physical examination indicators, we cannot definitively conclude that depression caused these changes, as the retrospective nature of the study does not allow us to rule out the possibility of reverse causation or other underlying factors. Secondly, since the study was conducted solely within a single medical center, caution is advised when generalizing and applying our results to broader populations or settings. The specific work environment, stressors, and population characteristics of this medical center might not fully represent those of other healthcare settings, thereby cautioning against broadly applying these findings to all medical staff. Thirdly, as the study is cross-sectional, it captures data at a single point in time, which precludes us from assessing the longitudinal effects of depression on physical health indicators. Without follow-up data, we cannot confirm whether the associations we observed persist over time or if they are influenced by transient factors. Fourthly, our exclusion criteria are not sufficient since there are other factors that might change blood indicator, such as unbalanced diabetes, history of psychiatric and neurological disorders, trauma, etc. Besides, measuring stress should be done through assessing the cortisol level in the appropriate day time. To address these limitations, future research should incorporate the measurement of cortisol levels at appropriate times of the day to more accurately assess physiological stress and consider prospective cohort studies that follow medical staff over time to better understand the directionality of these relationships and the potential causal pathways. Additionally, expanding the study to include multiple centers with diverse populations would enhance the generalizability of the findings and provide more robust evidence for the observed associations.

In summary, our study revealed a significant association between depression level and several physiological indicators in healthcare professionals, highlighting the need for targeted mental health strategies. Healthcare institutions should incorporate regular mental health evaluations, develop personalized support programs, and implement preventive health monitoring of relevant biomarkers. In order to enhance the health and overall well-being of medical personnel, it is imperative to conduct multi-center and prospective investigations with larger cohorts to validate the veracity of our findings. Future studies should expand on our findings by conducting longitudinal research to establish causal relationships between depression and physiological changes. This could include exploring the temporal dynamics of depression and physical health indicators over time, as well as investigating the potential mediating roles of lifestyle factors, such as diet, physical activity, and sleep. Moreover, examining the efficacy of targeted interventions, such as antioxidant therapy or stress reduction programs, in modulating these physiological markers could provide actionable strategies to mitigate the impact of depression in healthcare settings. Finally, future research should aim to explore the heterogeneity in responses to depression among healthcare workers, possibly considering genetic, environmental, and occupational differences that might influence these outcomes.

## Data Availability

The raw data supporting the conclusions of this article will be made available by the authors, without undue reservation.
